# Early essential newborn care for cesarean section newborns in China: study protocol for a multi-centered randomized controlled trial

**DOI:** 10.1186/s13063-022-06615-z

**Published:** 2022-08-19

**Authors:** Xueyin Wang, Xiaosong Zhang, Howard Lawrence Sobel, Zhao Li, Juan Juan, Huixia Yang

**Affiliations:** 1 Department of Obstetrics and Gynecology, Peking University First Hospital, No. 1 Xi’anmen Street, Beijing, 100034 China; 2 Maternal, Child Health and Quality and Safety, World Health Organization Regional Office for the Western Pacific, Manila, Philippines

**Keywords:** Early essential newborn care, Delayed cord clamping, Skin-to-skin contact, Breastfeeding, Cesarean section

## Abstract

**Background:**

Early essential newborn care (EENC) is a package of evidence-based and cost-effective interventions delivered around birth mainly including delayed cord clamping, immediate and sustained skin-to-skin contact, and early initiation of exclusive breastfeeding. EENC is proven effective in promoting breastfeeding and improving women’s and newborns’ health. However, there is little evidence on implementation of EENC on newborns born by cesarean section in China. Therefore, the objective of this study is to assess the effectiveness of EENC intervention on rates of exclusive breastfeeding and early initiation of breastfeeding for cesarean section newborns in China.

**Methods:**

This is a multi-centered, randomized controlled trial conducted in 4 tertiary hospitals in China. A total of 720 eligible women who will receive cesarean section are being randomized into four groups: control group (*n*=180), intervention group 1 (skin-to-skin contact for 30 min, *n*=180), intervention group 2 (skin-to-skin contact for 60 min, *n*=180), and intervention group 3 (skin-to-skin contact for 90 min, *n*=180). The control group will receive routine care, whereas the intervention groups will receive EENC with different duration of skin-to-skin contact. Demographic characteristics, clinical information, and breastfeeding outcomes will be collected. The primary outcome is rates of exclusive breastfeeding and early initiation of breastfeeding, and the secondary outcomes include maternal and neonatal morbidity and admissions.

**Discussion:**

This study will provide evidence of the impact of EENC on improvement of breastfeeding outcomes and maternal and neonatal health for cesarean section newborns in China, and evidence-based recommendation to inform optimal duration of skin-to-skin contact for cesarean deliveries. The results of this study have potential to inform national-level guidelines and policy-making for optimizing EENC implementation for cesarean section newborns.

**Trial registration:**

Chinese Clinical Trial Registry ChiCTR2100048997. Retrospectively registered on 19 July 2021

**Supplementary Information:**

The online version contains supplementary material available at 10.1186/s13063-022-06615-z.

## Administrative information

**Table:** 

**Title**	Early essential newborn care for cesarean section newborns in China: study protocol for a multi-centred randomized controlled trial
**Trial registration**	Retrospectively registered on Chinese Clinical Trial Registry on July 19, 2021 (ChiCTR2100048997).
**Protocol version**	Version 1.0; October 25, 2019
**Funding**	This study was supported by the UNICEF Regular Resources and Save the Children Hong Kong Unrestricted Funds.
**Name and contact information for the trial sponsor**	UNICEF, 12 Sanlitun Lu, Chaoyang District, Beijing, China; Save the Children, 2-2-52 Jianwai Diplomatic Compound, Chaoyang District, Beijing, China
**Role of sponsor**	The sponsor is not involved in study design, data collection and analysis, interpretation, manuscript preparation and submission for publication.

## Background

Deaths before 5 years of age have been estimated to 5.3 million globally in 2018, 47% of which occurred in the neonatal period (the first 28 days of life) [1]. In China, the neonatal mortality rate has been estimated at 4.5‰, accounting for nearly half of the under-five deaths [2]. To reduce preventable neonatal morbidity and mortality and improve women’s and newborns’ health, World Health Organization (WHO) and the United Nations Children’s Fund (UNICEF) jointly developed the Action Plan for Healthy Newborn Infants in the Western Pacific Region (2014–2020) which focused on delivery of early essential newborn care (EENC) [3]. EENC is a package of simple evidence-based and cost-effective interventions delivered around birth including thoroughly drying a newborn immediately after birth, delayed cord clamping, immediate and sustained skin-to-skin contact (SSC), initiating exclusive breastfeeding, and routine care (newborn eye care, vitamin K_1_, immunizations, weighting, and examinations) [3, 4]. Implementation of EENC has been demonstrated to increase rates of exclusive breastfeeding at discharge, 3 months and 6 months of age, and reduce neonatal intensive care unit admissions, admissions with hypothermia and sepsis [5, 6].

Accumulating evidence suggested that SSC contributed to increased exclusive breastfeeding rates on discharge, at 6 months and lower rate of transfer of newborns to the neonatal intensive care unit, regardless of mode of delivery [7–9]. In addition, babies born by cesarean section who received prolonged SSC had similar rates of exclusive breastfeeding at discharge as those born vaginally [4]. WHO recommends SSC for 1 h after birth which was consistent with implications of the Cochrane review [10, 11]. However, a recent cross-sectional observational study from the Western Pacific Region Early Essential Newborn Care Working Group reported a strong dose-response relationship between the duration of SSC and early initiation of breastfeeding through 90 min after birth [4]. Thus, the optimal duration of SSC needs further investigation.

Delayed cord clamping is another important procedure of EENC. A previous randomized controlled trial indicated that delayed cord clamping under cesarean section was associated with lower amount of postpartum bleeding, and increased hemoglobin and hematocrit in newborn heel blood [12]. Compared to those without delayed cord clamping, newborns with delayed cord clamping had decreased mean estimated maternal blood loss and transfusions and reduced prevalence of newborn anemia [13].

In 2016, EENC for vaginal deliveries was introduced in 6 hospitals in China, and afterwards scaled up to 18 counties in four provinces in Western China for pilot study [14, 15]. The study found that exclusive breastfeeding at discharge increased from 43 to 73%, and the average length of the first breastfeeding and the proportion of newborns receiving immediate SSC and prolonged SSC for more than 90 min were significantly increased [14]. To date, studies for EENC focused on newborns born by vaginal delivery in China, while there is limited evidence regarding implementation of EENC on cesarean section newborns. Furthermore, one-in-three (34.9%) births occur by cesarean section in China [16], and most of these babies might be deprived of the benefits of EENC. Therefore, the purpose of this study is to assess the effect of EENC intervention on breastfeeding outcomes and maternal and neonatal health for cesarean section newborns in comparison with the routine care in China. This study will be a parallel-group randomized trial, and participants will be allocated into 4 groups (1 control group and 3 intervention groups according to the duration of SSC) at a 1:1:1:1 ratio.

## Methods/ design

### Study design

**Fig. 1:**
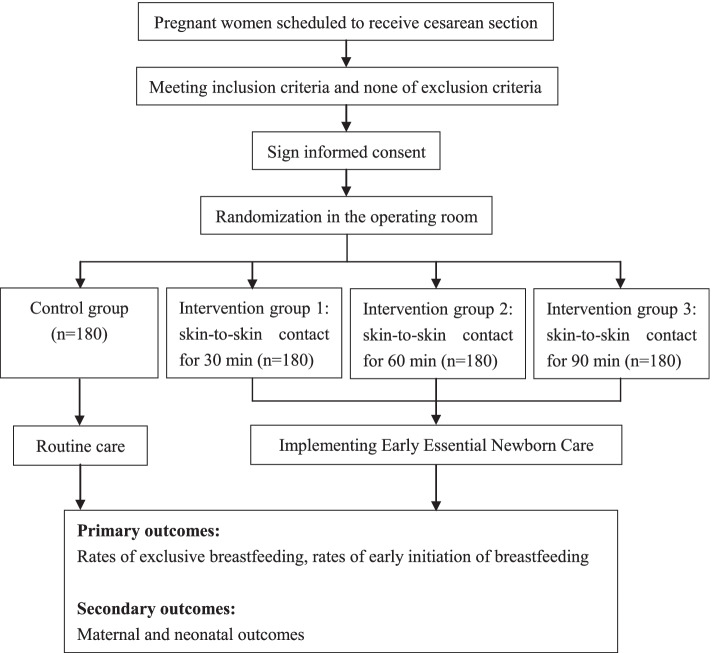
Flow chart of the study

This study is a multi-centered, randomized, open-labeled, superiority trial which is conducted at four tertiary hospitals in four provinces in China: Beijing, Sichuan, Shaanxi, and Ningxia. Four tertiary hospitals involved in our study are listed in Table 1. The study aims to compare the difference of early initiation of breastfeeding rates and exclusive breastfeeding rates among 4 different groups of mother and newborn pairs with full-term elective cesarean section (control group; intervention group 1: SSC for 30 min; intervention group 2: SSC for 60 min; and intervention group 3: SSC for 90 min). A total of 720 eligible pairs of mothers and neonates will be recruited and allocated into 4 groups at a 1:1:1:1 ratio. Figure 1 presents an explicit flow chart of this study. This clinical trial follows the guidelines for randomized clinical trials (SPIRIT checklist (Additional file 1)) [17]. The schedule of enrolment, interventions, and assessments are shown in Table 2.

**Table 1:** Study sites involving in this multi-centered randomized controlled study

Study sites	Location	Role
Peking University First Hospital	Beijing	Management and participant
Sichuan Provincial Hospital for Women and Children	Sichuan	Participant
Shaanxi Provincial Maternal and Child Health Care Hospital	Shaanxi	Participant
General Hospital of Ningxia Medical University	Ningxia	Participant

**Table 2:**
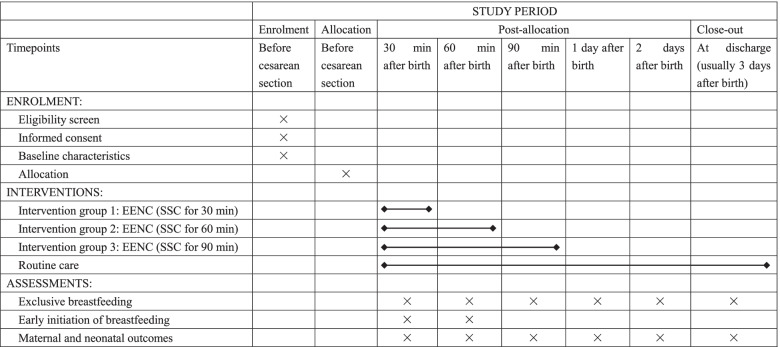
Schedule of enrolment, interventions, and assessments of this study

### Study participants

Among pregnant women who will receive cesarean delivery, eligible women and their newborns who meet the inclusion criteria and none of the exclusion criteria will be recruited in this study.

#### Inclusion criteria

Inclusion criteria are as follows:

1. Gestational weeks ≥ 37 weeks
2. Elective cesarean section
3. Singleton pregnancy
4. Epidural anesthesia, or subarachnoid block anesthesia, or combined spinal-epidural anesthesia
5. Able to provide written informed consent

#### Exclusion criteria

Exclusion criteria are as follows:

1. Serious pregnancy complications such as placenta previa, placenta accreta spectrum, and eclampsia
2. Neonatal complications such as neonatal asphyxia and neonatal birth defects
3. Unable to breastfeed
4. Difficulty in undergoing skin-to-skin contact
5. Infectious diseases such as hepatitis B, syphilis, and HIV

### Randomization and blinding

The randomization sequences are generated by the biostatistician using the random number sequence generator function in SAS software version 9.2 (SAS Institute, Cary, NC), with stratification by study sites. Participants will be assigned a randomization code according to their sequential numbers and randomly allocated into one of four groups with a 1:1:1:1 ratio in the operating room. Due to the nature of EENC interventions, blinding of participants, obstetricians, nurses/midwives, and intraoperative research staff is infeasible.

### Recruitment

In four study sites, obstetricians and midwives introduce the contents, advantages, and precautions of EENC to pregnant women and their families at prenatal visits and before delivery, including delayed cord clamping, immediate and continuous SSC, and early initiation of breastfeeding, so that pregnant women and their families could understand and cooperate in the implementation of EENC. Also, participants and their families will be educated to breastfeeding and early identification of neonatal risk signs (including breathing and skin color) and health care contents for newborns (including bathing, umbilical care, and vaccination). Among pregnant women who will receive cesarean section, eligible women will be identified and informed of procedures of EENC before cesarean section by obstetricians and midwives, and given the choice of participation on voluntary basis on the day of scheduled elective cesarean delivery. After informed consent is signed, research staff will randomly assign participants into one of four groups with a pre-generated randomization code. In addition to introducing the advantages and benefits of EENC to pregnant women and their families that have mentioned above, there will be no specific plans to promote participant retention. During skin-to-skin contact, midwives and researchers will emphasize the advantages and benefits of EENC and keep encouraging participants in order to help them to complete this process. All of the participants will be free to withdraw from the study at any time if they wish.

### Intervention and control groups

**Fig. 2:**
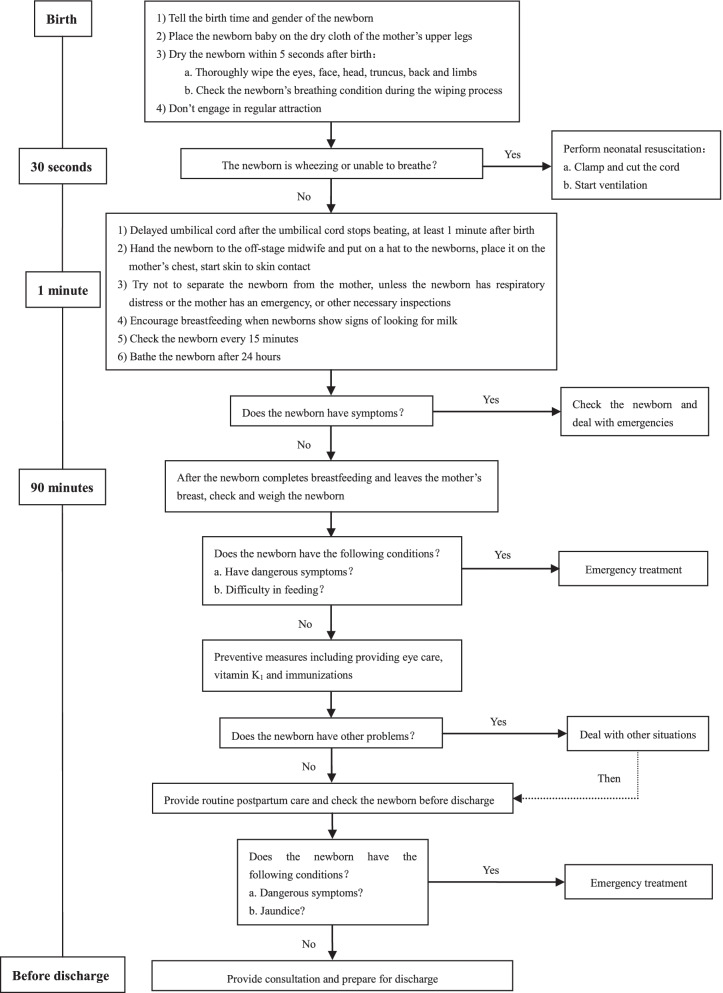
Flow chart of early essential newborn care for cesarean section

Pregnant women will be randomly assigned into one of four groups. The control group will receive only routine care (newborn eye care, vitamin K_1_, immunizations, weighing, and examinations) after birth, whereas the intervention group will receive EENC including delayed cord clamping, immediate and sustained SSC, initiating exclusive breastfeeding, and routine care. The flow chart of EENC for cesarean section is showed in Fig. 2. Delayed cord clamping is defined as cutting umbilical cord until cord pulsations have ceased (approximately 1–3 min after birth) [18]. Immediate and sustained SSC is regarded as placing of the naked baby prone on the mother’s bare chest immediately after birth without separation [10]. Pairs of mothers and newborns in the intervention group 1, intervention group 2, and intervention group 3 will perform SSC for 30 min, 60 min, and 90 min, respectively.

### Study outcomes

#### Primary outcomes

The primary outcome is exclusive breastfeeding rates at hospital discharge. Exclusive breastfeeding is defined as feeding only breastmilk with no other food or fluids except medications since birth [4]. Postnatal women will be asked the breastfeeding status of their baby (exclusively breastfeeding [fed only breast milk with the exception of water], main breastfeeding [fed breast milk and water], mixed feeding [fed breast milk, formula milk and water]) since the birth of their baby through the time of the interview at discharge. Another primary outcome is early initiation of breastfeeding defined as the completion of the first breastfeed by a newborn within an hour after birth [19].

#### Secondary outcomes

The secondary outcomes include maternal (postpartum hemorrhage, maternal hemoglobin at discharge) and neonatal outcomes (neonatal intensive care unit admission, neonatal ward admission, neonatal weight at discharge [usually 3 days after birth], neonatal transcutaneous bilirubin at discharge).

### Sample size calculation

We calculated the sample size based on the rate of early initiation of breastfeeding, which is one of the primary outcomes in this study. According to our pilot investigation and previous literature [4, 20], the rate of early initiation of breastfeeding is 40% in the control group (receiving routine care), and the rates in the intervention group 1 (receiving EENC with SSC for 30 min), intervention group 2 (receiving EENC with SSC for 60 min), and intervention group 3 (receiving EENC with SSC for 90 min) are supposed to be 60%, 70%, and 80%, respectively. Accounting for multiple comparisons (SSC for 30 min vs. routine care, SSC for 60 min vs. routine care, SSC for 90 min vs. routine care, SSC for 30 min vs. SSC for 60 min, SSC for 30 min vs. SSC for 90 min, SSC for 60 min vs. SSC for 90 min), a level of significance is set to be 0.008 (0.05/6) by using Bonferroni adjustments. For 80% power, the sample size required for each of the control group and the intervention group 1 is 152 participants; the sample size required for each of the control group and the intervention group 2 is 64 participants; the sample size required for each of the control group and the intervention group 3 is 34 participants. The largest sample size group (*n*=152) is selected as the number of recruitment in each group. Considering 15% of loss to follow-up, the number of participants in each group is finally set to be 180 and adds up to a total of 720 participants. One hundred eighty participants will need to be recruited in each of four hospitals, which means that each of the hospital will recruit 45 participants in the control group, intervention group 1, intervention group 2, and intervention group 3, respectively. The sample size calculation was performed using PASS 2011 software (NCSS, Kaysville, UT, USA).

### Analysis plan

Baseline characteristics of participants will be presented as mean (standard deviation) for continuous variables and number (percentage) for categorical variables. Data will be analyzed on an intention-to-treat basis to compare primary and secondary outcomes in four groups. The chi-square test or one-way analysis of variance will be used to compare the characteristics and rates of exclusive breastfeeding and early initiation of breastfeeding, and maternal and neonatal outcomes among the 4 groups (control group, intervention group 1, intervention group 2, and intervention group 3). For missing data, we will use multiple imputation to obtain complete datasets. All the statistical analyses will be conducted using SPSS 20.0 statistical software (SPSS Inc., Chicago, Illinois). A two-sided *P* value <0.05 indicated significance.

## Data management and monitoring

The data will be stored on h6world platform (h6world.cn). The research team will be given password to access this platform and obtain the data for analysis and interpretation. The data are monitored and audited annually by the Biomedical Research Ethics Committee of Peking University First Hospital. The data monitoring committee is independent from the sponsor and has no competing interests, and it will ensure that data management will be conducted according to the study protocol.

To ensure the confidentiality of the study data, each study participant will receive a unique identification number that will not be created by using their identifiable information. Only the research team will have access to the study data, and the data will not be shared with others.

## Plans for communicating important protocol modifications to relevant parties

Amendments to the study protocol will be communicated to the Biomedical Research Ethics Committee of Peking University First Hospital, and the research team. Participants to be recruited will be given a modified version of the study protocol before informed consent.

## Dissemination

The research team has the right to publish and is responsible for the results. All the collaborators are contributing significantly to the research, and the successful publication is credited to them equally. At the end of this study, we are expected to publish one or more scientific manuscripts in peer-reviewed journals. We also plan to present our results at national and international conferences through oral or poster presentations for further dissemination.

## Discussion

In China, EENC during vaginal deliveries has been performing since 2016 and contributed to increased rates of exclusive breastfeeding and improved maternal and neonatal health [4, 6, 15], while the evidence during cesarean deliveries remains lacking. The objective of this multi-centered randomized controlled study is to evaluate the effectiveness of EENC for cesarean section newborns on exclusive breastfeeding rates and early initiation of breastfeeding rates in China.

To the best of our knowledge, this study is the first multi-centered randomized controlled trial with relatively large sample size to determine the impact of EENC on improvement of breastfeeding outcomes and maternal and neonatal health for cesarean section newborns in China. We will recruit eligible women from four tertiary hospitals in four provinces that have been providing EENC during vaginal deliveries and accumulated abundant experience in EENC practices. All obstetricians, midwives, and researchers involved in implementation of EENC in four study sites were coached by national facilitators and passed post-coaching evaluations. Another strength of this study is that we will examine associations between the duration of SSC and breastfeeding performance, which will provide evidence-based recommendation to inform optimal duration of SSC for cesarean deliveries. The results of this study have potential to inform national-level guidelines and policy-making for optimizing EENC implementation for cesarean section newborns.

There are several limitations in this study. First, we currently focus on healthy mother and newborn pairs. Further studies will be needed among low-risk and high-risk mothers and newborns. Second, all study sites are tertiary-level hospitals with specialized obstetricians and midwives and advanced medical devices to ensure safety of mothers and newborns. In future studies, we plan to extend EENC implementation for cesarean section newborns to all levels of hospitals.

In conclusion, this is the first multi-centered randomized controlled study in China to assess the impact of EENC on improvement of breastfeeding outcomes and explore the optimal duration of SSC among newborns born by cesarean section. Our findings will enable making evidence-informed guidelines and policies.

## Trial status

The recruitment started in January 2021 and is expected to be completed in December 2021.

## Supplementary Information

**Additional file 1.** SPIRIT Checklist for this study.

## Data Availability

The data will be available from the corresponding author on reasonable request.

## Abbreviations

EENC: Early essential newborn care
SSC: Skin-to-skin contact
WHO: World Health Organization
UNICEF: United Nations Children’s Fund
